# Carbon Nanotube-, Boron Nitride-, and Graphite-Filled Polyketone Composites for Thermal Energy Management

**DOI:** 10.1021/acsomega.2c07924

**Published:** 2023-05-23

**Authors:** Yoldas Seki, Mehmet Mete Tokgöz, Ferhat Öner, Mehmet Sarikanat, Lutfiye Altay

**Affiliations:** † Faculty of Science, Dokuz Eylul University, Buca, Izmir 35160, Turkey; ‡ İzmir Eğitim SağlıkSanayiYatırım A.Ş., Turgutlu, Manisa 45400, Turkey; § Mechanical Engineering Department, Ege University, Bornova, Izmir 35040, Turkey

## Abstract

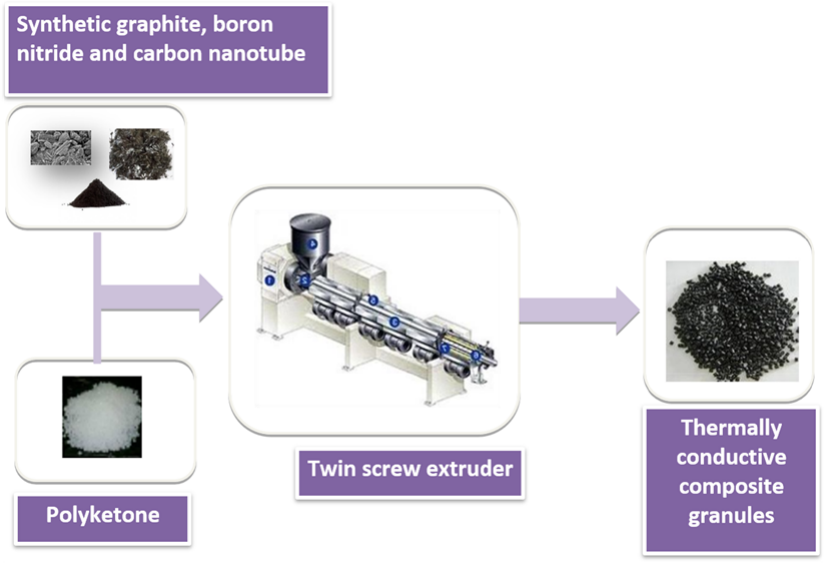

In order to improve the thermal conductivity of 30 wt % synthetic graphite (SG)-filled polyketones (POKs), conductive fillers such as multiwall carbon nanotubes (CNTs) and hexagonal boron nitride (BN) were used in this study. Individual and synergistic effects of CNTs and BN on 30 wt % synthetic graphite-filled POK on thermal conductivity were investigated. 1, 2, and 3 wt % CNT loading enhanced the in-plane and through-plane thermal conductivities of POK-30SG by 42, 82, and 124% and 42, 94, and 273%, respectively. 1, 2, and 3 wt % BN loadings enhanced the in-plane thermal conductivity of POK-30SG by 25, 69, and 107% and through-plane thermal conductivity of POK-30SG by 92, 135, and 325%. It was observed that while CNT shows more efficient in-plane thermal conductivity than BN, BN shows more efficient through-plane thermal conductivity. The electrical conductivity value of POK-30SG-1.5BN-1.5CNT was obtained to be 1.0 × 10^–5^ S/cm, the value of which is higher than that of POK-30SG-1CNT and lower than that of POK-30SG-2CNT. While BN loading led to a higher heat deflection temperature (HDT) than CNT loading, the hybrid fillers of BNT and CNT led to the highest HDT value. Moreover, BN loading led to higher flexural strength and Izod-notched impact strength values than CNT loading.

## 1 Introduction

The use of polymers in the applications of electronic packaging or encapsulations, satellite devices, and many more electronic devices may be insufficient to dissipate heat and deform less.^1^ The development of cooling systems is also essential in order to expand the lifetime of the electronic goods. Heat sinks are very popular cooling devices that can be designed in many shapes and from materials. Since polymers have generally lower thermal properties compared to metals and ceramics, development related to thermal properties is needed. $163 million is disbursed per year because 55% of the reported failures related to electronic devices stem from overheating.^2^ Lower coefficient of thermal expansion, higher thermal conductivity as well as lightweight are desired properties for this use.^3^ If suitable thermal conductive properties are gained, plastics can be very good alternative materials for heat sinks as they are being flexible, easy to fit in, and cost-friendly.^4^

It is known that aliphatic polyketones (POKs) are polymers derived from olefin monomers and carbon monoxide. They possess excellent chemical resistance, wear resistance, low gas permeability, and mechanical properties such as toughness. These intrinsic properties make POK a good alternative for polyamides, syndiotactic polystyrene, and polyesters such as polyethylene terephthalate and polybutylene terephthalate, and polyoxymethylene. Thus, POK can be considered as a good material for the electronic market and gear applications.^4,5^ In order to improve the thermal conductivity of POK, graphite, more especially expanded graphite, which merits special interest because of its abundant availability at a relatively low cost and lightweight when compared to other carbon allotropes, can be used.^6^ However, the low through-plane thermal conductivity of natural graphite sheet is a disadvantage.^7^ Aside from graphite, multiwall carbon nanotube (CNT) and hexagonal boron nitride (BN), the well-known thermal conductive fillers, have a wide thermal conductivity range of 1950–5000 and up to 600 W/mK, respectively.^8^ High-dimensional thermal conductive fillers offer a low interfacial area so that ineffectiveness of the thermal conductive fillers can be avoided. CNT might be a good example to establish a 3D structure in the composite.^9^ Formulations consisting of hybrid filler compositions and structures such as carbon nanotube–hexagonal boron nitride, carbon nanotube–graphene, and carbon nanotube–silver flake are found to be very efficient to obtain thermal conductive and superior dielectric materials.^8,9^ MWCNT was also preferred to be examined for its synergistic effect with other conductive fillers such as reduced graphene oxide,^10^ carbon fiber,^11^ and both conductive carbon black and graphene nanoplatelets.^12^

Kim and Kim investigated the synergistic effect of BN and MWCNT as a core–shell structure in the PPS matrix and found a negligible change in thermal conductivity.^13^ 10-BN/2-MWCNT and 20-BN/1-MWCNT led to an increase from 0.24 to 0.44 Wm^–1^ K^–1^ and 0.9 Wm^–1^ K^–1^, respectively. It can be said that BN and MWCNT had no synergetic effect on the thermal conductivity of the PPS matrix. TabkhPaz et al. also studied the synergistic effect of BN and MWCNT on the PS matrix and observed enhanced results.^14^ Low concentrations as 1.55 vol % of both CNT and BN yielded 290% increase in thermal conductivity. In that study, the alignment of the MWCNT was also investigated.^14^

In our trials, it was observed that POK, having more than 30 wt % of synthetic graphite, caused extruding problems such as strand breaks during production. In order to improve the thermal conductivity of 30 wt % synthetic graphite (SG)-filled POK, other conductive fillers such as carbon nanotubes and hexagonal boron nitride were used for the synergistic effects in this study. Individual and synergistic effects of carbon nanotubes and hexagonal boron nitride on 30 wt % synthetic graphite-filled POK on thermal conductivity were investigated. It was also determined how synergists affected the electrical conductivity of POK-SG composites. Moreover, the effect of synergists on thermal conductivity, impact, and mechanical properties of these composites were discussed.

## 2 Materials and Methods

### 2.1 Materials

POK M330 as a polymer matrix was supplied from Hyosung Chemical Corporation. HeBoFill 511 which is a pure boron nitride powder with a d_50_ of 10 microns was used in this study. Synthetic graphite (SG), TIMREX KS 44, and CNTs (MWCNTs: purity, 92%; outside diameter, 8–10 nm) were obtained from IMERYS Graphite & Carbon (Switzerland) and Nanografi (Türkiye), respectively.

### 2.2 Compounding Process

A twin-screw extruder, 27 mm in diameter and with 12 zones (Leistritz 27 MAXX), was used to produce composite granules. Test specimens were prepared from composite granules by an injection molding machine (Bole model BL90EK). In this study, thermal conductive fillers, SG, BN, and CNT, were used at 30, 1–3, and 1–3 wt % fractions in POK-based composites, respectively. In our trials, it was observed that more than 30 wt % of synthetic graphite caused extruding problems during production.

### 2.3 Thermal Conductivity

Thermal conductivity (*k*, W/mK) of POK-based composites was calculated from thermal diffusivity (α, m^2^/s), density (ρ, g/cm^3^), and specific heat (*C*_p_, J/kgK) values by using eq 1
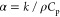
1

The specific heat values of the composites were measured by using differential scanning calorimetry. The measurement of thermal diffusivity values of POK-based composites was carried out with a Discovery Xenon Flash DXF 200 system (TA Instruments Inc.) with respect to ASTM E 1461 standard.

### 2.4 Thermogravimetric Analysis

Degradation temperatures of BN- and CNT-filled polyketone materials were measured by using TA Instrument’s TGA Q50. The analyses were performed at the rate of 10 °C/min under nitrogen atmosphere up to 800 °C.

### 2.5 Differential Scanning Calorimetry

Melting and crystallization temperatures of the BN-, CNT-, and SG-filled POK composites were measured at the heating rate of 10 °C/min under nitrogen atmosphere. Heat capacities of the samples were measured by DSC Q20 by using modulated DSC.

### 2.6 Density

Density measurement of the POK-based composites was conducted by using Densimeter MD-200S. Average of three tests was recorded for the density values.

### 2.7 Thermomechanical Analysis

The thermal expansion coefficients (CTEs) of POK and its composites were determined by using a TMA400 system (TA Instruments). Specimens (10 × 5 × 3 mm) were heated from 20 to 120 °C at a rate of 5 °C min^–1^. The measurements were carried out in expansion mode.

### 2.8 Heat Deflection Temperature

HDT-A values of POK and its composites were obtained according to ISO 75 standards under a specific load of 1.8 MPa using a Coesfeld Vicat/HDT testing device.

### 2.9 Electrical Conductivity

Electrical conductivities of POK and its composites were obtained by using a Keithley digital DC source meter.

### 2.10 Flexural Properties

The flexural properties of POK and its composites were tested on a universal testing machine (Hegewald & Peschke Inspect 20 universal testing machine) by using a three-point bending test at the rate of 2 mm/min according to ISO 178 standards.

### 2.11 Impact Properties

Izod-notched impact tests of POK and its composites were carried out by using pendulum impact testing machine (Instron––CEAST 9050 Impact Pendulum) according to ISO 180 standards. The Izod-notched impact strength, expressed as mean ± standard deviation, was analyzed using Student’s *t* test for the calculation of the significance level of the data. Differences were taken as statistically significant at *P* ≤ 0.05.

### 2.12 Scanning Electron Microscopy

SEM observation of the samples was performed by using a scanning electron microscope (Carl Zeiss 300VP, Germany) operated at 7.5 kV. Prior to the SEM analysis, the surfaces of the specimens were coated with a thin layer of gold via a plasma sputtering apparatus.

## 3 Results and Discussion

### 3.1 Thermal Conductivity

**Figure 1:**
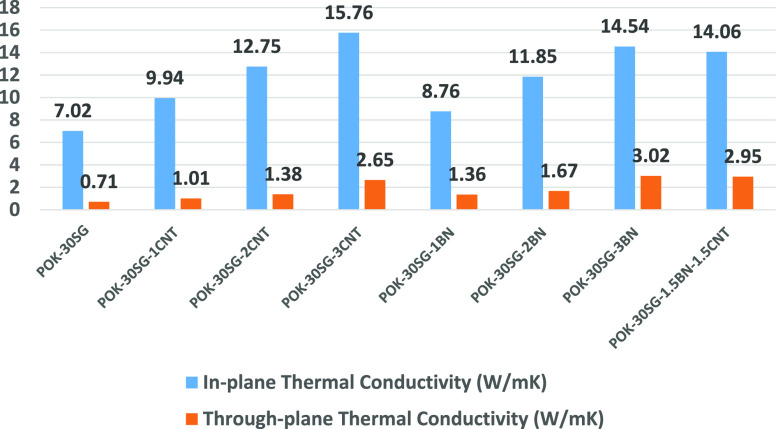
Thermal conductivities of POK-based composites.

The in-plane and through-plane thermal conductivities of POK-based composites are presented in Figure 1. The thermal conductivity of POK was obtained to be 0.21 W/mK. The effect of CNT, BN, and CNT–BN on the thermal conductivities of POK-30SG can be clearly seen in Figure 1. 1, 2, and 3 wt % CNT loadings led to 42, 82, and 124% increases in the in-plane thermal conductivity of POK-30SG and 42, 94, and 273% in the through-plane conductivity of POK-30SG, respectively. It is seen that CNT addition caused a higher thermal conductivity in the through-plane direction. Increasing the weight fraction of CNT reduces the matrix region between CNTs in a composite and facilitates the interaction between CNTs, which in turn contributes to the increase of thermal conductivity.^15^ 1, 2, and 3 wt % BN loadings led to 25, 69, and 107% and 92, 135, and 325% increases in the in-plane and through-plane thermal conductivities of POK-30SG, respectively. It can be reported that CNT loading caused higher thermal conductivity values in the in-plane direction compared to the BN addition. On the other hand, BN loading resulted in higher thermal conductivity values in the through-plane direction. At a low filler concentration region, BN platelets of larger size exhibited a higher thermal conductivity because smaller BN particles have larger interfacial areas which may act as thermally resistant junctions leading to phonon scattering.^16^ It is reported that BN particles with larger size (approx. 7–10 μm) could easily create the network structure of BN.^17^ When the thermal conductivity values of POK-30SG-3CNT, POK-30SG-3BN, and POK-30SG-1.5BN-1.5CNT were compared, POK-30SG-3CNT and POK-30SG-3BN have higher in-plane and through-plane conductivities, respectively. Moreover, for the need of higher through-plane conductivity, instead of POK-30SG-3CNT, POK-30SG-1.5BN-1.5CNT can be preferred.

### 3.2 Thermogravimetric Analysis

**Figure 2:**
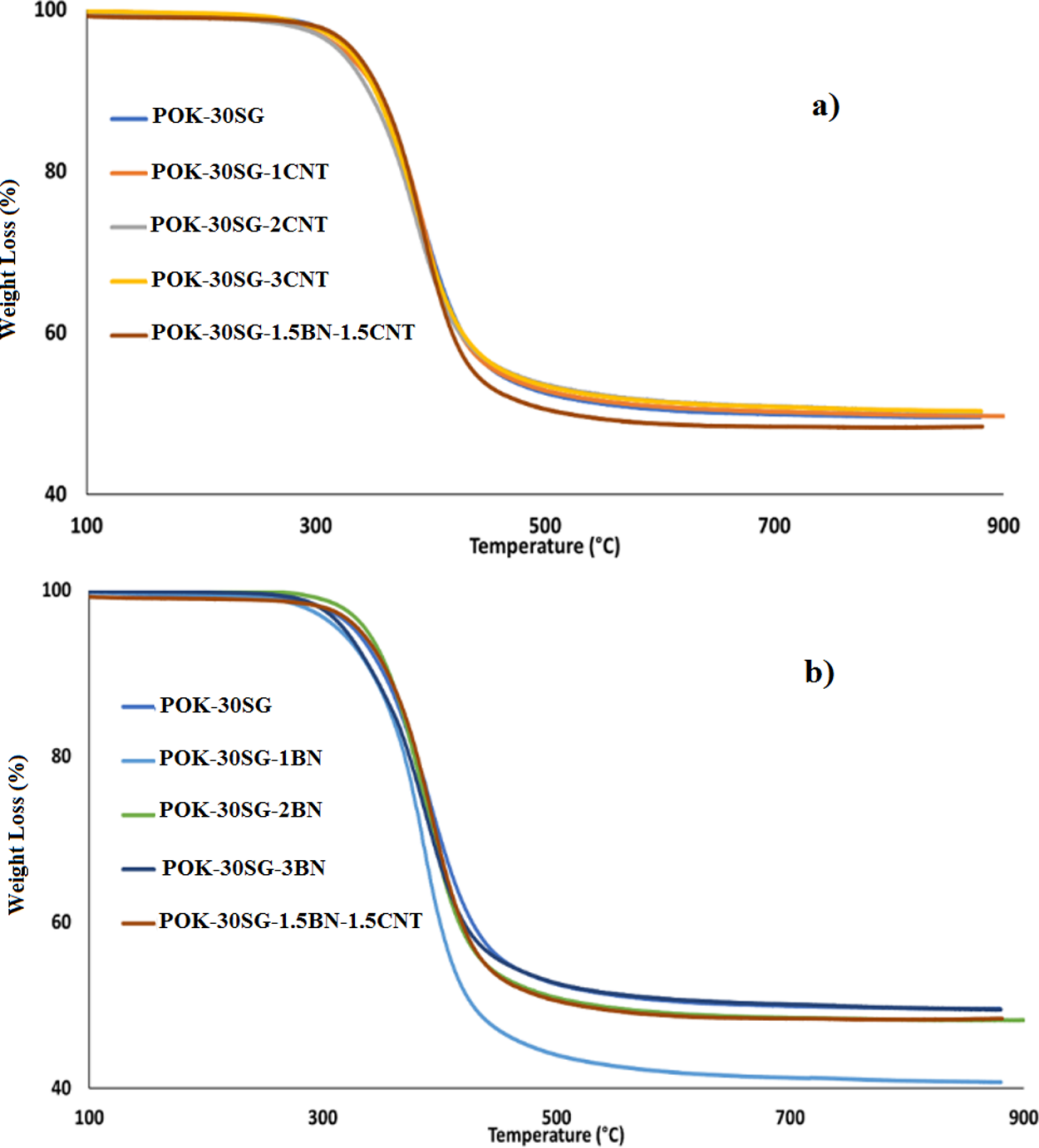
Thermograms of 30 wt % synthetic graphite-filled POK composites containing (a) CNT and (b) BN.

Thermogravimetric analyses (TGA) have a critical role in the thermal conductivity property. The TGA thermograms of samples are given separately in Figure 2. From Figure 2, temperature values at 5% mass loss were obtained and summarized in Table 1. As can be seen from Table 1, the temperature values at 5% mass loss of POK-30SG, POK-30SG-1CNT, POK-30SG-2CNT, POK-30SG-3CNT, POK-30SG-1BN, POK-30SG-2BN, POK-30SG-3BN, and POK-30SG-1.5BN-1.5CNT were obtained to be 329, 323, 320, 329, 316, 338, 320, and 333 °C, respectively. POK-30SG-1CNT, POK-30SG-2BN, and POK-30SG-1.5BN-1.5CNT have higher temperature values at 5% mass loss than POK-30SG. It can be said that it is possible to increase the thermal stability of POK-30SG by adding CNT, BN, and the combination of BN–CNT.

**Table 1:** Temperature Values at 5% Mass Loss of the Samples

sample	*T* (°C) at 5% mass loss
POK-30SG	329
POK-30SG-1CNT	323
POK-30SG-2CNT	320
POK-30SG-3CNT	329
POK-30SG-1BN	316
POK-30SG-2BN	338
POK-30SG-3BN	320
POK-30SG-1.5BN-1.5CNT	333

**Figure 3:**
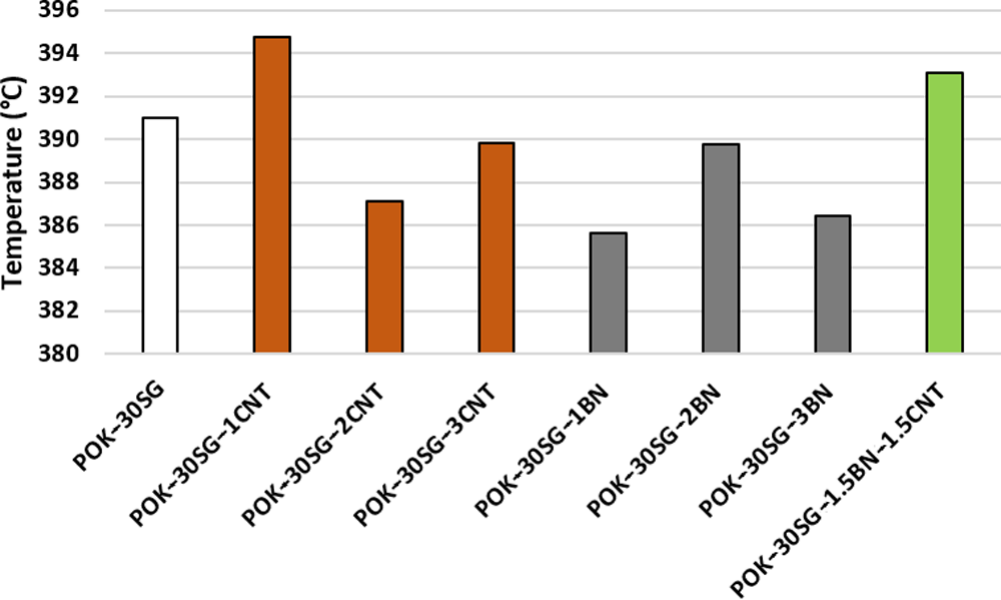
*T*_max_ values of the samples.

The analyses for only synthetic graphite (POK-30SG), in addition to synthetic graphite and individual carbon nanotube (POK-30SG-1CNT, POK-30SG-2CNT, and POK-30SG-3CNT)- and boron nitride (POK-30SG-1BN, POK-30SG-2BN, and POK-30SG-3BN)-filled polyketone composites were performed with respect to their maximum degradation temperatures (*T*_max_). Figure 3 represents the maximum degradation temperatures of POK-30SG-based composites. The temperature range covers a slight narrow band of 385–395 °C.

The highest degradation temperatures were observed in the case of POK-30SG-1CNT. This result is in good agreement with the temperature values at 5% mass loss presented in Table 1. Then, further loading of CNT led to a fluctuation in the *T*_max_ value, first with a dramatic decrease and then a slight increase. An inverse trend was observed, as in the case of boron nitride-filled POK-30SG samples. The blend of CNT and BN contributed a good thermal stability, as indicated in Figure 3. It is observed that 3 wt % of the blend of both inclusions displayed superior thermal performance compared to the 3 wt % of CNT and BN, separately.

### 3.3 DSC Analysis

**Figure 4:**
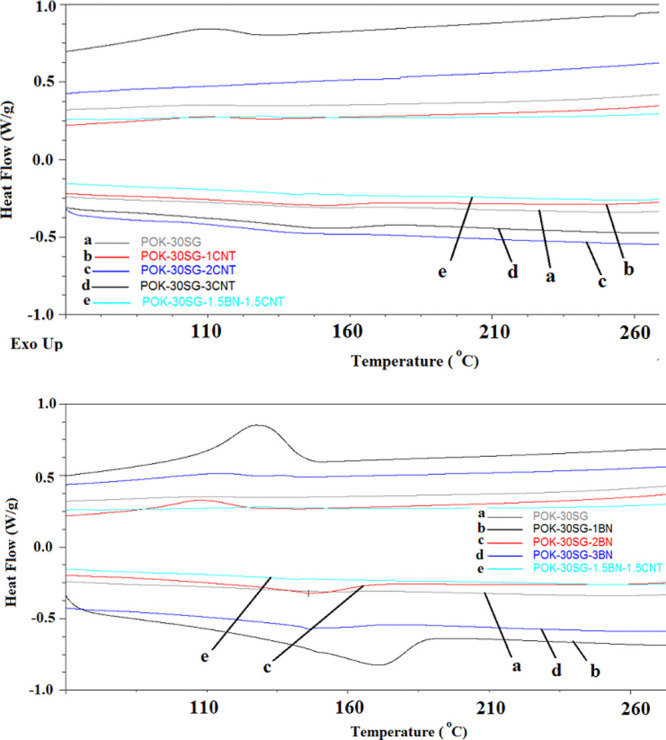
DSC curves of POK-30 SG-based composites.

The synergetic effects of BN and CNT thermal conductive fillers on 30 wt % synthetic graphite-filled POK are investigated with respect to crystallization and melting temperatures. The DSC curves of the samples are represented in Figure 4 and summarized in Table 2. The summary includes the degree of crystallization (*X*_c_), melting enthalpy (Δ*H*_m_), and crystallization enthalpy (Δ*H*_c_). The degree of crystallization is calculated with respect to eq 2(18)
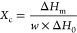
2where *w* is the weight fraction of POK in the composite, and Δ*H*_0_ is the melting enthalpy of the fully semicrystalline POK material, which is obtained as 226 J/g.^19^

**Table 2:** Thermal Properties of POK Composites with Various Fillers

samples	*T*_m_ (°C)	Δ*H*_m_ (J/g)	*X*_c_ (%)	*T*_c_ (°C)	Δ*H*_c_ (J/g)
POK-30SG	a	a	a	a	a
POK-30SG-1CNT	a	a	a	a	a
POK-30SG-2CNT	a	a	a	a	a
POK-30SG-3CNT	a	a	a	a	a
POK-30SG-1BN	172	12.2	35	129	15.4
POK-30SG-2BN	146	9.5	28	107	10.6
POK-30SG-3BN	146	6.2	18	113	4.3
POK-30SG-1.5BN-1.5CNT	140	0.4	1	130	0.6

a No obtained data available.

Unfortunately, CNT-filled POK-30SG composites yielded no melting and crystallization temperatures and thus no melting and crystallization enthalpies after differential scanning calorimetry analysis. DSC curves displayed amorphous-like behavior, as can be seen in Figure 4. However, a peak was yielded in the case of BN and hybrid structure. An increasing amount of BN resulted in a decrease in the melting point. However, a melting temperature stability was observed in BN fillings of 2 and 3 wt %. In the formulation consisting of hybrid fillers, the lowest melting temperature was obtained. Both the melting enthalpy and degree of crystallization displayed a decreasing trend with the increasing BN load, accordingly. The same trend was also observed in heat capacities. It can be summarized that increasing the amount of thermal conductive fillers yielded a lower temperature, thus lower energy, to melt the composite.

### 3.4 Density

**Figure 5:**
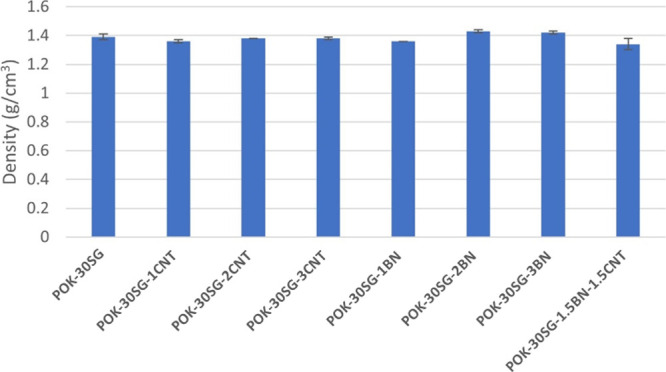
Density values of samples.

The density values of POK-based composites are shown in Figure 5. The density value of POK (1.24 g/cm^3^) increased to 1.39 g/cm^3^ when 30 wt % SG was added to the composites. The density of SG, CNT, and BN is relatively higher than that of POK (1.24 g/cm^3^); thus, the incorporation of these fillers into POK increased the density of the neat POK. It is observed that density values remained close to each other when single or hybrid combinations of fillers were used in this study. POK-30SG-2BN and POK-30SG-3BN composites have higher density than that of POK-30SG.

### 3.5 Heat Deflection Temperature

The HDT values of POK-based composites are given in Table 3. Although there was a small increase in the HDT for 1 wt % loading of CNT, there was a noticeable decrease in the HDT values at higher loadings (2 and 3 wt %) compared to the POK-30SG sample. It is known that the HDT-A test utilizes a constant load (1.8 MPa). From DSC analyses, it is observed that POK-SG composites containing CNT have no melting temperature, which indicates the disappearance of the crystal structure. It is expected that, above the glass-transition temperature, the crystals contribute to the load-bearing capability of the polymers. However, BN addition increased the HDT value of POK-30SG composites. Moreover, from Table 3, it is observed that POK-30SG composites containing BN have melting temperatures, which shows the crystal parts within the polymer. It is seen that when hybrid fillers were used in POK-based composites, the highest HDT value (155 °C) was obtained.

**Table 3:** HDT Values of POK-Based Composites

sample	HDT (°C)
POK-30SG	109
POK-30SG-1CNT	114
POK-30SG-2CNT	79
POK-30SG-3CNT	82
POK-30SG-1BN	122
POK-30SG-2BN	106
POK-30SG-3BN	146
POK-30SG-1.5BN-1.5CNT	155

### 3.6 Thermomechanical Analysis

The thermal expansion coefficients (CTE) of the POK-based composites prepared with SG, CNT, and/or BN are listed in Table 4.

**Table 4:** CTE Values of POK-Based Composites

sample	CTE (μm/m °C)
POK-30SG	105
POK-30SG-1CNT	100
POK-30SG-2CNT	95
POK-30SG-3CNT	91
POK-30SG-1BN	105
POK-30SG-2BN	97
POK-30SG-3BN	86
POK-30SG-1.5BN-1.5CNT	104

When Table 4 is examined, it is seen that the addition of single or hybrid CNT and BN resulted in lower CTE values in the composites. In addition, the obtained values showed that the decrease in CTE increased as the fraction of fillers increased, which caused a significant improvement in thermal properties. It has also been known that the CTE of a polymer composite depends on the orientation of the filler with respect to the direction of flow. One can say that the good alignment of CNT and/or BN in the direction of flow during the fabrication process is the main reason of reduced CTE values in POK composites.^20^

### 3.7 Electrical Conductivity

The electrical conductivity values of POK and its composites are presented in Table 5. The electrical conductivity of SG was obtained to be 8.9 × 10^–6^S/cm. As can be seen from Table 5, while CNT loading increased the electrical conductivity of POK-30SG, BN loading decreased the electrical conductivity of POK-30SG because of the electrical conduction nature of CNT^21^ and electrical insulation nature of BN,^22^ respectively. As the weight fraction of CNT increased, the electrical conductivity of POK-based composites increased due to the higher weight fractions of fillers interconnected to form a conductive continuous network.^23^ The electrical conductivity of POK-30SG-3CNT is larger, about 600 times, than that of POK-30SG. Moreover, as the weight fraction of BN increased, electrical conductivity decreased. The electrical conductivity value of POK-30SG-1.5BN-1.5CNT was obtained to be 1.0 × 10^–5^ S/cm, which is a higher value than that of POK-30SG-1CNT and lower than that of POK-30SG-2CNT.

**Table 5:** Electrical Conductivity Values of POK-Based Composites

sample	electrical conductivity (S/cm)
POK-30SG	8.9 × 10^–6^
POK-30SG-1CNT	3.5 × 10^–5^
POK-30SG-2CNT	9.2 × 10^–4^
POK-30SG-3CNT	5.4 × 10^–3^
POK-30SG-1BN	9.3 × 10^–6^
POK-30SG-2BN	1.2 × 10^–7^
POK-30SG-3BN	2.1 × 10^–7^
POK-30SG-1.5BN-1.5CNT	1.0 × 10^–5^

### 3.8 Flexural Properties

**Figure 6:**
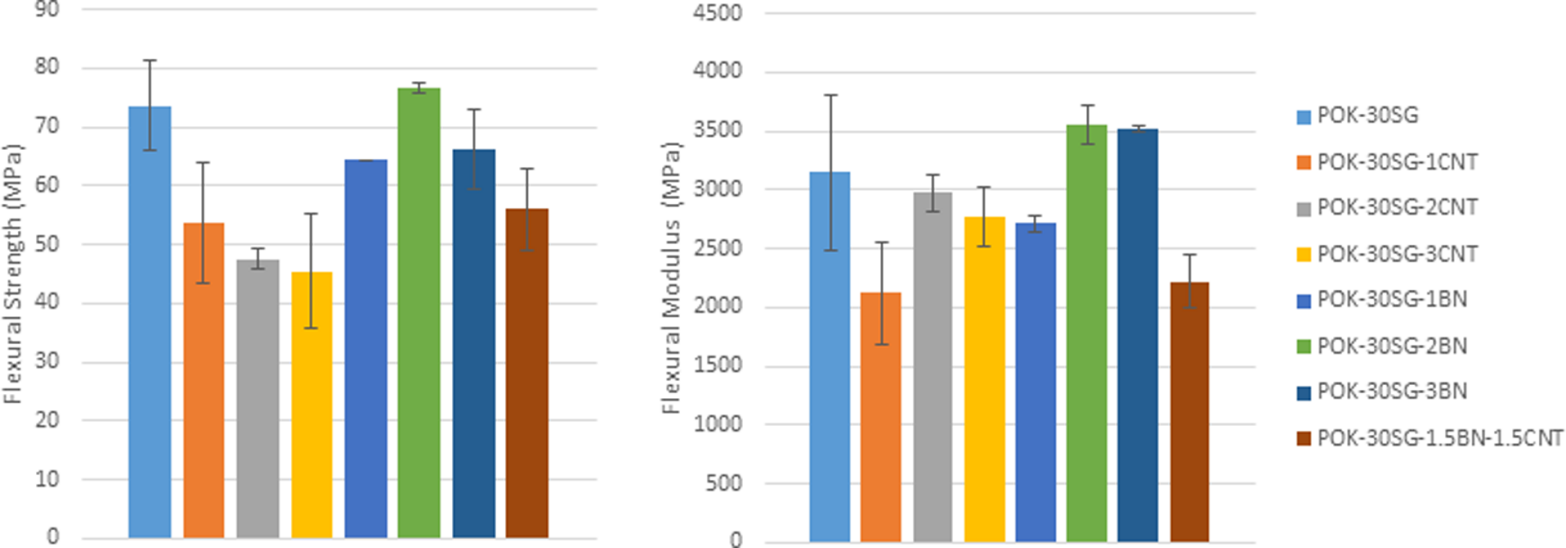
Flexural strength and modulus values of POK and its composites.

The flexural and flexural modulus values are shown in Figure 6. Flexural strengths of 73.6, 53.8, 47.5, and 45.5 MPa were obtained for PPS-30SG and 1, 2, and 3 wt % CNT-based POK, respectively. 1, 2, and 3 wt % CNT loadings decreased the flexural strength of POK-30SG. The flexural strength values of POK-30SG-1BN, POK-30SG-2BN, POK-30SG-3BN, and POK-30SG-1.5BN-1.5CNT were obtained to be 64.4, 76.8, 66.2, and 56.0 MPa, respectively. While 1 wt % BN loading slightly increased the flexural strength of POK-30SG, 2 and 3 wt % loadings decreased the flexural strength of POK-30SG. From the flexural modulus values, it is seen that BN loading led to higher values than CNT loading. The lower flexural strength and flexural modulus of the CNT-added POK-30SG composite, compared to the BN-loaded POK-30SG composite, may be due to the poor dispersion of CNT in POK-30SG. It was noted that well-dispersed CNT composites demonstrated improved mechanical properties than poorly dispersed CNT composites in the polymer system.^24^ It is worth stating that a uniform dispersion and a good interfacial bonding are important for load transfer to the CNTs across the CNT–matrix interface.^25^

### 3.9 Impact Properties

**Figure 7:**
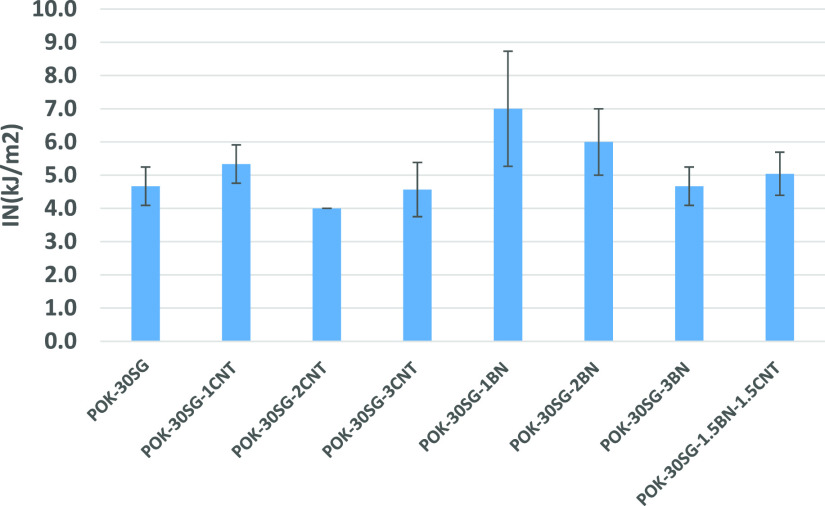
IN values of POK and its composites.

Izod-notched impact strength (IN) values of POK-based composites are shown in Figure 7. The Izod-notched impact strength of POK was obtained to be 9.8 kJ/m^2^. IN values of 4.7, 5.3, 4.0, and 4.6 kJ/m^2^ were obtained for the POK-30SG and 1, 2, and 3 wt % CNT-filled SG-based POK composites, respectively. 1 wt % CNT loading led to improvement in the IN value of POK-30SG. The IN values of POK-30SG-1BN, POK-30SG-2BN, POK-30SG-3BN, and POK-30SG-1.5BN-1.5CNT were obtained to be 7.0, 6.0, 4.7, and 5.0 kJ/m^2^, respectively. 1 and 2 wt % BN loadings led to about 49 and 28% increase in the IN value of POK-30SG. From Figure 7, it is seen that POK-30SG-1.5BN-1.5CNT is superior to POK-30SG-3BN and POK-30SG-3CNT in terms of IN values. When P values (>0.05) are considered, there is no significant difference in the IN values between POK-30SG and others (POK-30SG-3CNT, POK-30SG-3BN, and POK-30SG-1.5BN-1.5CNT). According to Kirmani et al., filler dispersion alone is not a sufficient condition to improve the impact strength of the composites. However, filler type and processing conditions are also important for the impact strength of composites.^26^ Ghoshal et al. indicated that significant property improvements can be seen at a relatively low CNT concentration of 1 wt % by tailoring the interphase between the carbon nanotubes and the polymer matrix,^27^ which is compatible with our results in terms of impact strength. Pai et al. emphasized that the higher the amount of BN was incorporated in the system, the lower the impact strength of the composites and the more severe aggregation occured.^28^

### 3.10 SEM Analysis

**Figure 8:**
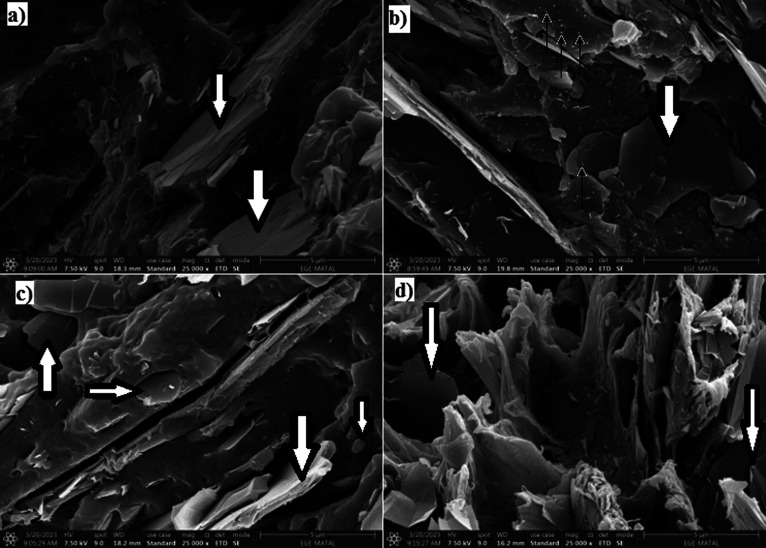
SEM images of samples. (a) PBT-30SG, (b) PBT-30SG-3BN, (c) PBT-30SG-3CNT, and (d) PBT-30SG-1.5BN-1.5CNT.

The SEM micrographs of samples are presented in Figure 8. Graphite sheets can be seen in Figure 8a–d (with a large arrow). Since the weight fraction of BN and CNT is fairly low as compared to SG, CNT and BN particles cannot be seen clearly in Figure 8d. BN particles in Figure 8b and CNT particles in Figure 8c are shown with arrows. It seems that the CNT distribution is not homogeneous on the POK matrix surface. However, BN distribution is more homogeneous. The sizes of BN particles are smaller than those of CNT particles.

## 4 Conclusions

The effect of CNT and BN on the thermal conductivity of POK-30SG was investigated. 1, 2, and 3 wt % CNT loadings increased the in-plane thermal conductivity of POK-30SG by 42, 82, and 124% and through-plane conductivity of POK-30SG by about 42, 94, and 273%, respectively. However, and 1, 2, 3 wt % BN loadings increased the in-plane thermal conductivity of POK-30SG by 25, 69, and 107% and the through-plane thermal conductivity of POK-30SG by 92, 135, and 325%. This indicates that while CNT loading causes higher thermal conductivity values in the in-plane direction, BN loading causes higher thermal conductivity values in the through-plane direction. In terms of electrical conductivity, it was observed that the electrical conductivity of POK-30SG-3CNT is larger about 600 times than that of POK-30SG. Moreover, as the weight fraction of BN was increased, electrical conductivity decreased. The electrical conductivity value of POK-30SG-1.5BN-1.5CNT was obtained to be 1.0 × 10^–05^ S/cm, which is a higher value than that of POK-30SG-1CNT and lower than that of POK-30SG-2CNT. It can be reported that in order to obtain better thermal and electrical conductivities of POK, the combination of SG-CNT and SG-BN can be used.
